# Improvement in the management of chronic obstructive pulmonary disease following a clinical educational program: results from a prospective cohort study in the Sicilian general practice setting

**DOI:** 10.1038/s41533-018-0077-7

**Published:** 2018-03-23

**Authors:** Rosarita Ferrara, Valentina Ientile, Carlo Piccinni, Alessandro Pasqua, Serena Pecchioli, Andrea Fontana, Umberto Alecci, Riccardo Scoglio, Francesco Magliozzo, Sebastiano Emanuele Torrisi, Carlo Vancheri, Patrizio Vitulo, Giovanna Fantaci, Carmen Ferrajolo, Mario Cazzola, Claudio Cricelli, Achille Patrizio Caputi, Gianluca Trifirò

**Affiliations:** 1 Unit of Clinical Pharmacology, Academic Hospital G. Martino, Messina, Italy; 2 Department of Medical and Surgical Sciences, University of Bologna, Bologna, Italy; 3 Health Search, Italian College of General Practitioners and Primary Care, Florence, Italy; 4 Unit of Biostatistics, IRCCS Casa Sollievo della Sofferenza, San Giovanni Rotondo, Foggia, Italy; 5 Italian Society of General Practice (SIMG), Catania, Italy; 6 Regional Referral Center for Rare Lung Diseases, University – Hospital “G. Rodolico”, Department of Clinical and Experimental Medicine, University of Catania, Catania, Italy; 7 Pulmonology Unit, Department for the Treatment and Study of Cardiothoracic Diseases and Cardiothoracic Transplantation, IRCCS – ISMETT (Istituto Mediterraneo per i Trapianti e Terapie ad Alta Specializzazione), Palermo, Italy; 8 Epidemiologic Observatory – Sicilian Regional Department of Health, Palermo, Italy; 9 Department of Experimental Medicine, University of Campania “Luigi Vanvitelli”, Napoli, Italy; 10 Department of Systems Medicine, University of Rome Tor Vergata, Rome, Italy; 11 Department of Biomedical Sciences, Dentistry and Functional and Morphologic Imaging, University of Messina, Messina, Italy

## Abstract

Chronic obstructive pulmonary disease (COPD) is a chronic inflammatory disorder of the lungs associated with progressive disability. Although general practitioners (GPs) should play an important role in the COPD management, critical issues have been documented in the primary care setting. The aim of this study was to evaluate the effectiveness of an educational program for the improvement of the COPD management in a Sicilian general practice setting. The effectiveness of the program, was evaluated by comparing 15 quality-of-care indicators developed from data extracted by 33 GPs, at baseline vs. 12 and 24 months, and compared with data from a national primary care database (HSD). Moreover, data on COPD-related and all-cause hospitalizations over time of COPD patients, was measured. Overall, 1,465 patients (3.2%) had a registered diagnosis of COPD at baseline vs. 1,395 (3.0%) and 1,388 (3.0%) over time (vs. 3.0% in HSD). COPD patients with one spirometry registered increased from 59.7% at baseline to 73.0% after 2 years (vs. 64.8% in HSD). Instead, some quality of care indicators where not modified such as proportion of COPD patients treated with ICS in monotherapy that was almost stable during the study period: 9.6% (baseline) vs. 9.9% (after 2 years), vs. 7.7% in HSD. COPD-related and all-cause hospitalizations of patients affected by COPD decreased during the two observation years (from 6.9% vs. 4.0%; from 23.0% vs. 18.9%, respectively). Our study showed that educational program involving specialists, clinical pharmacologists and GPs based on training events and clinical audit may contribute to partly improve both diagnostic and therapeutic management of COPD in primary care setting, despite this effect may vary across GPs and indicators of COPD quality of care.

## Chronic lung disease: Education improves disease management

An education program for doctors covering chronic lung disease diagnosis and management improves elements of patient care in Italy. Gianluca Trifirò at the University of Messina, Italy, and co-workers followed a cohort of 33 family doctors and 1,465 patients with chronic obstructive pulmonary disease (COPD) in Sicilian primary care for two years, comparing quality of care for patients before and after implementing the education program. The training, led by lung specialists and clinical pharmacologists, updated doctors with the latest information on COPD diagnosis, medication and disease management, and trained them in spirometry test interpretation. The team assessed the value of the program using quality-of-care indicators and asked each doctor to re-evaluate patients’ COPD diagnosis. Their results showed improvement in some areas, including spirometry testing, alongside a reduction in hospital admissions over the two-year period.

## Introduction

Chronic obstructive pulmonary disease (COPD) is an irreversible chronic inflammatory disorder of the lungs, characterized by the different combination of chronic bronchitis and emphysema. It is caused by exposure to noxious agents, such as tobacco smoke.^1^ COPD, if not treated promptly and accurately, is characterized by progressive pulmonary airflow reduction resulting in disability and respiratory failure, ultimately leading to increased risk of morbidity and mortality.^2^ Younger patients may benefit from lung transplantation.^3^

The World Health Organisation estimates that 65 million people around the world suffer from COPD, which has been predicted to become the third leading cause of death worldwide by 2030.^4^

In Italy, the prevalence of COPD ranges from 1.5 to 5.0%, with higher values in older people, in men and in Southern Italy.^5^ The physicians mainly involved in diagnosis and management of COPD are pulmonologists and general practitioners (GPs); the latter ones act as gatekeepers of the national health system by providing the citizens with healthcare services as drug prescriptions and requests of specialists’ visits and hospitalizations thus playing a major role in the management of chronic diseases such as COPD.

An Italian observational study reported that GPs prescribe frequently drugs targeting obstructive airway diseases without taking into account COPD severity as recommended in treatment guidelines, therefore a large number of COPD patients receiving inappropriate therapy.^6^ Another Italian survey documented a poor agreement between ‘the Global Initiative For Chronic Obstructive Lung Disease’ (GOLD) recommendations^7^ and current clinical practice in ambulatory care.^8^ Moreover, another Italian epidemiologic study showed that spirometry results were not available for 40% of COPD patients,^9^ which was in line with other European observational studies.^10,11^ These findings evidence the urgency to develop strategies aimed at improving COPD management, especially in primary care setting. For this purpose various educational programs have been proven as a valid tool to ameliorate the quality of care of COPD patients, in several countries.^12–14^ Similarly, this prospective study was aimed to evaluating the effectiveness of an educational program promoting the best practices for diagnosis, management and treatment of COPD in a Sicilian general practice setting.

## Results

### Demographic, clinical and therapeutic characteristics of COPD patients

Out of a total sample of 46,326 inhabitants registered in the lists of 33 GPs participating to the project, 1,465 (3.2%) were automatically identified from electronic archives as patients with a COPD diagnosis at baseline (before the intervention) whereas 12 and 24 months after the intervention the COPD diagnosis was confirmed for 1,395 (3.0%) and 1,388 (3.0%) patients, respectively (Table 1). By 31 December 2015, patients with a COPD diagnosis who were registered in the GPs’ participating to HSD were 31,691 (3%).

**Table 1:** Demographic, clinical and therapeutic characteristics of patients with COPD diagnosis who are cared by General Practitioners (GPs) participating to the study evaluation before and after educational intervention vs. Italian general population (Health Search—IMS Health Longitudinal Patient Database: HSD)

	Pre-intervention	Post intervention		P value^a^	Italian GPs database - HSD	P value^b^
	Baseline	Follow-up at 12 mo.	Follow-up at 24 mo.	Baseline vs. FU at 24 mo.		HSD vs. FU at 24 mo.
	No. 1,465	No. 1,395	No. 1,388	No. 1,025 patients^c^	No. 31,691	
*Gender*						
Male	929 (63.4)	892 (63.9)	900 (64.8)	—	18,455 (58.2)	<0.001
Female	536 (36.6)	503 (36.1)	488 (35.2)	—	13,236 (41.7)	
*Age*—*Median (Q1*–*Q3)°*	74.0 (65.0–81.0)	74.0 (65.0–81.0)	74.0 (65.0–81.0)	—	74.0 (65.0–81.0)	0.364
<45	29 (2.0)	27 (1.9)	24 (1.7)	—	1108 (3.5)	<0.001
45–54	89 (6.1)	82 (5.9)	68 (4.9)	—	2025 (6.4)	0.025
55–64	221 (15.1)	216 (15.5)	223 (16.1)	—	4677 (14.7)	0.179
65–74	406 (27.6)	402 (28.8)	400 (28.8)	—	8739 (27.5)	0.310
75–84	517 (35.3)	480 (34.4)	478 (34.5)	—	10,109 (31.9)	0.047
≥85	203 (13.9)	188(13.5)	195 (14.0)	—	5033 (15.8)	0.066
*Smoking habit* ^d^						
Smoker	84 (5.7)	59 (4.2)	64 (4.6)	0.414	8664 (27.3)	<0.001
Former smoker	670 (45.7)	701 (50.3)	706 (50.9)	0.005	8267 (26.1)	<0.001
No smoker	366(25.0)	359 (25.7)	354 (25.5)	<0.001	8484 (26.7)	0.296
Unknown data	345 (23.6)	276 (19.8)	264 (19.0)	<0.001	6276 (19.8)	0.473
*BMI* ^d^						
Underweight (<18.5)	9 (0.6)	15 (1.1)	14 (1.0)	0.046	427 (1.3)	0.281
Normal Weight (18.5–24.9)	169 (11.5)	179 (12.8)	204 (14.7)	0.033	6623 (20.9)	<0.001
Overweight (25.0–29.9)	376 (25.7)	387 (27.7)	414 (29.8)	<0.001	8928 (28.1)	0.180
Obese (>29.9)	556 (38.0)	550 (39.5)	502 (36.2)	0.497	8427 (26.6)	<0.001
Unknown data	355 (24.2)	264 (18.9)	254 (18.3)	<0.001	7286 (22.9)	<0.001
*Co-morbidities* ^d^						
Hypertension	881 (60.1)	893 (64.0)	888 (64.0)	<0.001	21,219 (66.9)	0.021
Diabetes mellitus	419 (28.6)	437 (31.3)	432 (31.2)	<0.001	8312 (26.2)	<0.001
Osteoporosis	269 (18.4)	275 (19.7)	288 (20.8)	<0.001	7523 (23.7)	0.010
Anxiety and depression	336 (22.9)	340 (24.4)	358 (25.8)	<0.001	9855 (31.1)	<0.001
Dementia	83 (5.7)	104 (7.5)	112 (8.1)	<0.001	3151 (9.9)	0.021
Myocardial infarction	107 (7.3)	110 (7.9)	105 (7.6)	0.008	5021 (15.8)	<0.001
Heart Failure	150 (10.2)	137 (9.8)	146 (10.5)	<0.001	3012 (9.5)	0.208
Pneumonia	24 (1.6)	34(2.4)	40(2.9)	0.014	1161 (3.6)	0.127
*COPD pharmacological treatment* ^*e,f*^						
LABA + ICS	450 (30.1)	420 (30.1)	401 (28.9)	0.003	8,996 (28.4)	0.683
LAMA	347 (23.2)	395 (28.3)	368 (26.5)	0.324	8,089 (25.5)	0.408
ICS	335 (22.4)	353 (25.3)	325 (23.4)	0.848	6,048 (19.1)	<0.001
LABA + LAMA + ICS	96 (6.6)	107 (7.7)	103 (7.4)	0.758	3,552 (11.2)	<0.001
LABA	127 (8.7)	139 (10.0)	136 (9.8)	0.377	2,598 (8.2)	0.034
Xanthines	93 (6.4)	88 (6.3)	70 (5.0)	0.157	1,747 (5.5)	0.452
LABA + LAMA	34 (2.3)	48 (3.4)	71 (5.1)	0.001	297 (0.9)	<0.001
*Concomitant drugs* ^e^						
Antihypertensive drugs	1,034 (70.6)	992 (71.1)	990 (71.3)	0.003	21,830 (68.8)	0.054
Antibiotics	907 (61.9)	1,080 (77.4)	1,156 (83.3)	<0.001	16,267 (51.3)	<0.001
Systemic corticosteroids	439 (30.0)	460 (33.0)	459 (33.1)	0.008	8,079 (25.5)	<0.001
Beta blockers	351 (24.0)	361 (25.9)	384 (27.7)	0.001	8,365 (26.4)	0.293
Anti-thrombotic drugs	250 (17.1)	234 (16.8)	189 (13.6)	0.129	4,397 (13.8)	0.785
Immunosuppressive drugs	6 (0.4)	7 (0.5)	8 (0.6)	0.046	129 (0.4)	0.336

^a^*p*-values from McNemar’s Test (not performed for patient’s sex and age variables because patient’s sex cannot change from baseline whilst patient’s age obviously increase of 2 years exactly from baseline)

^b^*p*-values were calculated using Chi-square test while Student’s test was used for median values (comparisons between HSD vs. FU at 24 months)

^c^No. of patients with registrations both at baseline and after 24 months

^d^Any time

^e^In the last year; BMI: Body Mass Index; ICS: Inhaled Corticosteroids; LABA: Long Acting Beta Agonist; LAMA: Long Acting Muscarinic Antagonist

^f^Not mutually exclusive

In all three time points male subjects were >63% of the cohorts and the median age was 74 years old (q1–q3: 65.0–81.0), which is rather in line with HSD data (male subjects: 58.2%; median age: 74.0 years old (q1–q3: 65.0–81.0). Around 50% of COPD patients of both Sicilian GP network and HSD were current or former smokers, with a proportion of those who stopped smoking which was much higher in the intervention group than in HSD (45–50% vs. 26%) and which increased over time. Higher proportion of obese COPD patients was reported in Sicilian GPs’ network patients as compared to HSD (40% vs. 27%). The most commonly reported comorbidities as evaluated at the three time points were: hypertension (from 60.1% at baseline to 64.0% at the two FU visits vs. 66.9% in HSD; *p*-value Baseline vs. FU at 24 mo.: <0.001; *p*-value HSD vs. FU at 24 mo.: 0.021) and diabetes mellitus (28.6% vs. 31.2%; 26.2% in HSD; *p*-value Baseline vs. FU at 24 mo.: <0.001; *p*-value HSD vs. FU at 24 mo.: <0.001), followed by anxiety and depression (22.9% vs. 24.4–25.8%; 31.1% in HSD; *p*-value Baseline vs. FU at 24 mo.: <0.001; *p*-value HSD vs. FU at 24 mo.: <0.001), osteoporosis (18.4% vs. 19.7%-20.8%; 23.7% in HSD; *p*-value Baseline vs. FU at 24 mo.: <0.001; *p*-value HSD vs. FU at 24 mo.: 0.010) and heart failure (10.2%, 9.8–10.5%; *p*-value Baseline vs. FU at 24 mo.: <0.001; *p*-value HSD vs. FU at 24 mo.: 0.208).

Among patients in treatment with respiratory drugs evaluated in the previous year, the most frequently prescribed medications were: LABA + ICS (30.1, 30.1 and 28.9%; 28.4% in HSD; *p*-value Baseline vs. FU at 24 mo.: 0.003; *p*-value HSD vs. FU at 24 mo.: 0.683), LAMA alone (23.2% vs. 28.3 % vs. 26.5%; 25.5% in HSD; *p*-value Baseline vs. FU at 24 mo.: 0.324; *p*-value HSD vs. FU at 24 mo.: 0.408) and ICS alone (22.4, 25.3 and 23.4%; 19.1% in HSD; *p*-value Baseline vs. FU at 24 mo.: 0.848; *p*-value HSD vs. FU at 24 mo.: <0.001). This was followed by triple therapy (LABA/LAMA + ICS) in 6.6, 7.7 and 7.4% of COPD patients as registered in the three time periods while this percentage was slightly higher in HSD (11.2%). The use of LABA + LAMA slightly increased from 2.3% at baseline to 3.4 and 5.1% at follow-up periods, but was very low in HSD (0.9%) (*p*-value Baseline vs. FU at 24 mo.: 0.001; *p*-value HSD vs. FU at 24 mo.: <0.001). Anti-hypertensive drugs (70.6, 71.1 and 71.3%; *p*-value Baseline vs. FU at 24 mo.: 0.003), and antibiotics (61.9, 77.4 and 83.3%; *p*-value Baseline vs. FU at 24 mo.: <0.001) were the most frequently prescribed drugs other than respiratory medicines in the year prior the assessment, as confirmed in HSD (68.8 and 51.3%, respectively).

### Evaluation of the intervention effects on quality-of-care indicators

The COPD prevalence (indicator 1) among patients in charge of Sicilian GPs decreased from baseline (3.2%) to follow-up visits (3.0%). These estimates are in line with the prevalence from national HSD (3.0%).

As regards diagnostic process indicators, COPD patients with a spirometry examination registered lifetime (indicator 2) increased from 59.7% before intervention to 70.5 and 73.0%, respectively, after 12 and 24 months from the educational intervention. Among HSD cohort this percentage was 64.8%. By restricting the analysis only to smokers, the proportion of COPD patients with a spirometry lifetime increased from 70.5 to 77.7% and 81.0% over time; these values were higher than those recorded in HSD (65.3%). Moreover, analysing the patients with a spirometry test registration in the last year (indicator 4) this rate increased from 25.3 to 36.1% and 32.1% respectively, which is much higher as compared to what reported in HSD (13.6%). Also the proportion of COPD patients with a registration of BMI (indicator 6) and smoking habits (indicator 7) gradually increased from 75.8% (baseline) to 81.7% (24 months) and from 76.5% (baseline) to 81.0% (24 months), respectively. These percentages are in line with value recorded in HSD (76.4 and 80.2%).

**Fig. 1:**
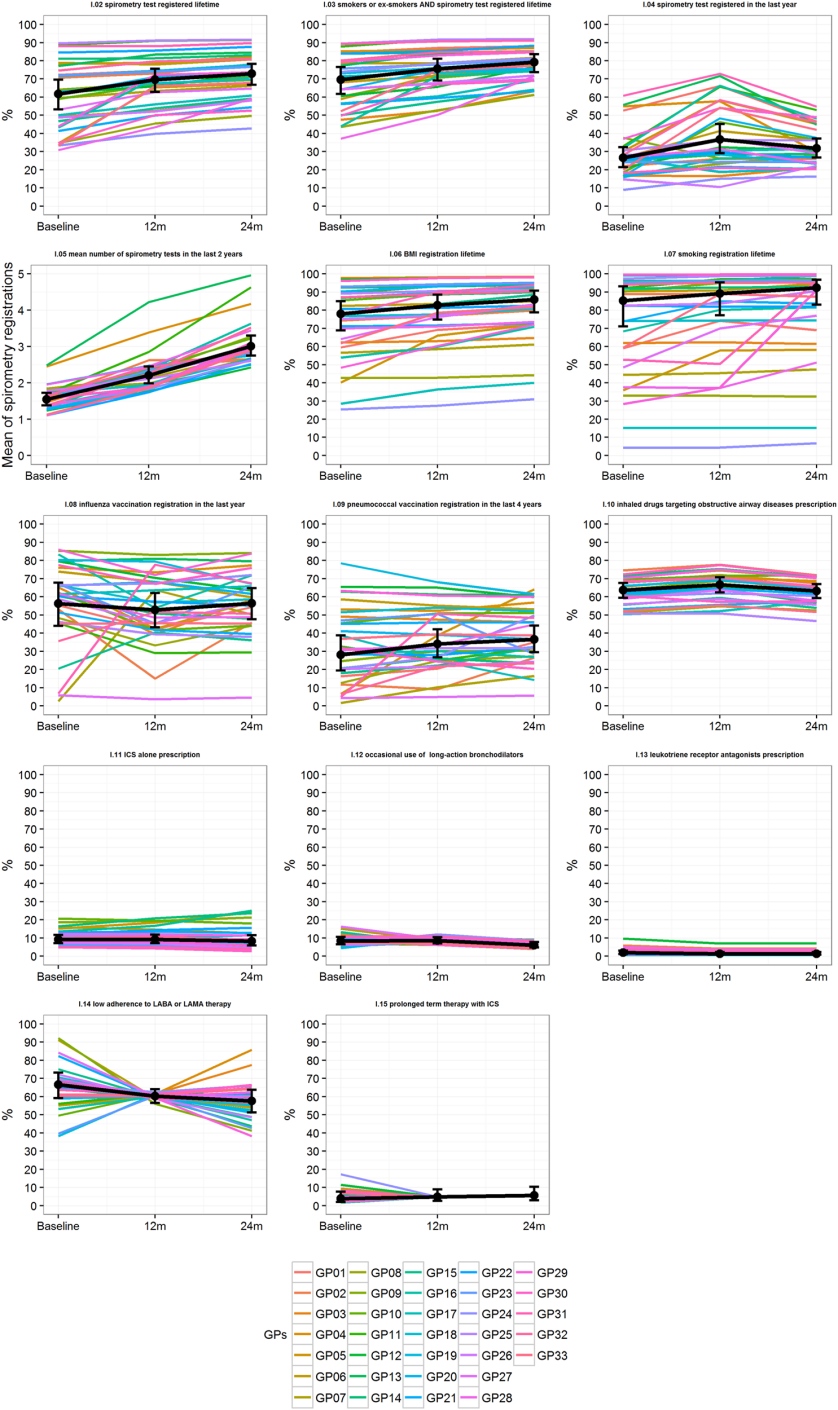
Longitudinal plots of the estimated (i.e., clustered-adjusted) proportions over time, along with profiles estimated for each GP. Error bars represented 95% confidence interval

The proportion of patients with influenza vaccination in the last year (indicator 8) slightly decreased from baseline (57.2%) to 12 months (55.1%) and 24 months (55.7%) after the intervention. This percentage was much higher in Sicilian GPs’ research network rather than in HSD (31.0%). Instead a slight increase was observed for the proportion of COPD patients receiving *Streptococcus pneumoniae* vaccination in the last four years (indicator 9) (32.6% vs. 34.8% and 35.1%), which is much higher than HSD (15.0%). Concerning pharmacological treatment indicators, COPD patients treated with any drug targeting obstructive airway diseases (indicator 10) increased from 61% at baseline to around 64% in the follow up visits at 12 and 24 months, which is higher than HSD (53.2%). The proportion of COPD patients treated with ICS in monotherapy (indicator 11) was almost stable during the study period and it was higher than what reported in HSD. Although the percentage of patients with occasional use of LABA and/or LAMA plus ICS or less (indicator 12) decreased from baseline (8.5%) to 24 months of follow-up (6.3%), it was always higher as compared to HSD (4.5%). The proportion of patients receiving anti-leukotrienes (indicator 13) decreased from baseline (2.3%) to follow-up periods (1.7 and 1.9%), in line with values recorded in HSD (1.8%). COPD patients with a low adherence to LABA and/or LAMA treatment (plus ICS or less) (indicator 14) decreased from 61.6% before intervention to 54.7% after 24 months, in line with HSD data (55.0%). The proportion of COPD patients in treatment with prolonged use of ICS (indicator 15) remained almost stable over time (5.8% at baseline vs. 5.1% at 24 months), but it was lower than what reported in HSD (9.3%). Such proportions were re-estimated using HGLMs for each indicator, separately, to account for the effect of clustering effect. Longitudinal plots of the estimated (i.e., clustered-adjusted) proportions over time were reported in Fig. 1, along with the estimated profiles for each GP. In addition, we reported the percentage for GPs attitude and behaviour for each specific indicator, at the three time points in Table 2. Benchmarking of GP- and time period-specific quality of care indicators are reported as Supplementary Material.

**Table 2:** Percentage of GPs with positive, neutral and negative behaviours for each specific indicator (row frequencies)

	Baseline			At 12 months			At 24 months		
Indicator	Negative	Neutral	Positive	Negative	Neutral	Positive	Negative	Neutral	Positive
2. Spirometry lifetime	14 (42.4)	6 (18.2)	13 (39.4)	10 (30.3)	14 (42.4)	9 (27.3)	11 (33.3)	13 (39.4)	9 (27.3)
3. Spirometry lifetime among smokers	12 (36.4)	10 (30.3)	11 (33.3)	10 (30.3)	13 (39.4)	10 (30.3)	9 (27.3)	14 (42.4)	10 (30.3)
4. Spirometry in the last year	11 (33.3)	15 (45.5)	7 (21.2)	14 (42.4)	7 (21.2)	12 (36.4)	12 (36.4)	11 (33.3)	10 (30.3)
5. Mean *n*. spirometry in the last 2 years	11 (33.3)	15 (45.5)	7 (21.2)	14 (42.4)	12 (36.4)	7 (21.2)	10 (30.3)	17 (51.5)	6 (18.2)
6. BMI registration lifetime	11 (33.3)	9 (27.3)	13 (39.4)	11 (33.3)	8 (24.2)	14 (42.4)	11 (33.3)	10 (30.3)	12 (36.4)
7. Smoking registration lifetime	13 (39.4)	9 (27.3)	11 (33.3)	12 (36.4)	10 (30.3)	11 (33.3)	12 (36.4)	11 (33.3)	10 (30.3)
8. Influenza vaccination in the last year	5 (15.2)	19 (57.6)	9 (27.3)	11 (33.3)	9 (27.3)	13 (39.4)	9 (27.3)	13 (39.4)	11 (33.3)
9. Pneumococcal vaccination in the last 4 years	9 (27.3)	12 (36.4)	12 (36.4)	12 (36.4)	9 (27.3)	12 (36.4)	11 (33.3)	8 (24.2)	14 (42.4)
10. Drugs targeting obstructive airway diseases in the last year	8 (24.2)	12 (36.4)	13 (39.4)	9 (27.3)	15 (45.5)	9 (27.3)	10 (30.3)	13 (39.4)	10 (30.3)
11. ICS in monotherapy in the last year	9 (27.3)	15 (45.5)	9 (27.3)	9 (27.3)	14 (42.4)	10 (30.3)	9 (27.3)	14 (42.4)	10 (30.3)
12. Occasional use of LABA and/or LAMA (±ICS) in the last year	8 (24.2)	19 (57.6)	6 (18.2)	3 (9.1)	27 (81.8)	3 (9.1)	6 (18.2)	22 (66.7)	5 (15.2)
13. Leukotriene receptor antagonists use in the last year	9 (27.3)	13 (39.4)	11 (33.3)	9 (27.3)	13 (39.4)	11 (33.3)	9 (27.3)	13 (39.4)	11 (33.3)
14. Low adherence to LABA and/or LAMA (±ICS)	5 (15.2)	20 (60.6)	8 (24.2)	0 (0)	32 (97.0)	1 (3.0)	6 (18.2)	19 (57.6)	8 (24.2)
15. Prolonged use of ICS	5 (15.2)	27 (81.8)	1 (3.0)	0 (0)	33 (100)	0 (0)	0 (0)	33 (100)	0 (0)

Legend: from indicator n.2 to n.10: ‘positive’, ‘neutral’ and ‘negative’ behaviours were referred to be above, within and below the mean of the estimated registrations proportion, respectively. From indicator n.11 to n.15: ‘positive’, ‘neutral’ and ‘negative’ behaviours were referred to be below, within and above the mean of the estimated registrations proportion, respectively

**Fig. 2:**
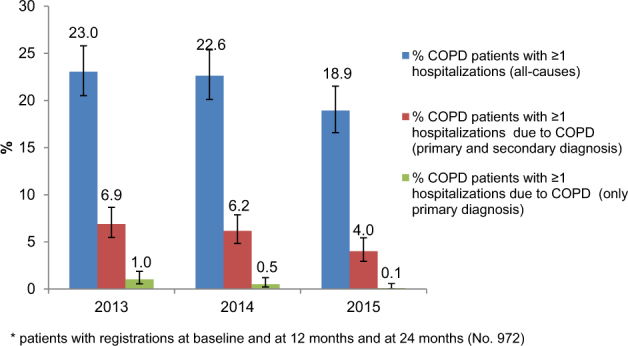
Percentage of COPD patients* who have been hospitalized due to COPD and all-causes and during the study period (2013–2015 years)

Concerning data from COPD patients being hospitalized, a decreasing trend was measured during the study years with respect to COPD-related only primary discharge diagnosis (1.0% vs. 0.1%), COPD-related both primary and secondary discharge diagnosis (6.9% vs. 4.0%), and all-cause hospitalization (23.0% vs. 18.9%) (Fig. 2).

## Discussion

This prospective, community-based study suggests an improvement in some of COPD management facets, thanks to the implementation of a complete educational program, comprising training events and clinical audit, in Sicilian primary care. In particular, the frequency of spirometry test registrations showed a marked increase during the study period, both in the overall COPD population and in smoker patients, in line with recommendations on COPD. Nevertheless, yet 27% of COPD patients did not receive any spirometry to confirm diagnosis even after the educational program thus confirming the challenge of performing spirometry test in all suspected COPD patients in routine care. This percentage is lower with respect to that showed by Han et al.^15^ where only 32% of patients with a new COPD diagnosis had undergone spirometry within the previous 2 years.

To increase awareness on the role of spirometry in primary care to diagnose appropriately COPD, relevant strategies have been proposed, as described in several European clinical studies.^12–14^ Educational programmes based on spirometry workshops or new spirometry delivery models with specifically trained health professionals might improve the correct diagnosis rate and the quality of spirometry testing. With this respect, Soler et al.^13^ showed that training GPs in spirometry can improve the rate of correct diagnosis from 56 to 89%. Our study demonstrated through audit of real-world data, a substantial improvement in some diagnostic process indicators, such as BMI or smoking registration, implicated in a correct management of COPD. Conversely, the slightly decreasing of influenza vaccination registration might due to the ‘Fluad case in Italy’, during the study period.^16^

Our analysis also confirms also discrepancies between current care and recommended best practice, referring to therapeutic treatment. Although, our results showed that COPD patients treated with bronchodilators slightly increased during the study period (indicator 10), a large percentage (36.7%) of patients remained undertreated, similarly to findings by Price et al.^17^ and by Barrecheguren et al.^18^

In COPD treatment, international guidelines recommend ICS use only in association with bronchodilators, especially in advanced COPD stages.^7^ Despite this recommendation was underlined during the educational program, our findings showed that the proportion of patient treated with ICS in monotherapy (without any bronchodilators) remained similar before and after the intervention (indicator 11) as this may partly due to treatment of other respiratory acute diseases such as sinusitis. On the other hand, this inappropriate use of ICS may be due to misdiagnosis between COPD and other chronic respiratory diseases, such as asthma and pulmonary fibrosis. Previous studies showed that inaccurate diagnosis of COPD leads to inappropriate pharmacological management.^19,20^ Another critical issue in the COPD treatment is the long-term adherence to the prescribed therapy.^21,22^ The proportion of COPD patients with low adherence to long-acting bronchodilators (±ICS) (indicator 14) decreased during the study period. In a recent analysis, Di Martino et al. found that about 29% of patients were adherent to long-acting bronchodilators; however, adherence increased for patients discharged from pneumology wards.^23^ Interestingly, we observed over the observation years a decrease of the COPD-related and all-cause hospitalization rates for COPD patients of enrolled GPs which may be the result of the observed improvement of several process indicators, in line with data from a recent regional report (http://www.asppalermo.org/Archivio/regolamenti/Obiettivi%20strategici%202016-2017/Report_Esiti_Sicilia_16.pdf).

This study has several strengths. First, this study showed that electronic medical data, properly extracted, anonymized and analyzed from GP’s archives, may provide important information on the chronic disease management. In addition, prospective assessment of the COPD patients allowed diagnosis validation. Second, we evaluated COPD management in a Sicilian primary care setting through objective assessment over time of 15 process indicators as well as outcome indicators through linkage with claims databases. Third, to evaluate the effectiveness of educational intervention program we evaluated not only historical controls but also a cohort of GPs distributed all over Italy and that are part of HSD.^24^

Some limitations of the study warrant cautions. First, this study was carried out using data collected from a Sicilian primary care setting. Participating GPs are already involved in a regular clinical research activity and this may explain why Sicilian GPs enrolled in the study performed, with respect to several COPD process indicators, already better at baseline as compared to GPs that are part of HSD. For the same reason, our study results cannot be fully generalized to GPs practicing all over Italy or outside Italy. Nevertheless, this study in general provides relevant insight on the potential effect of educational interventions on COPD management in primary care, especially regarding improvement of COPD diagnosis through training session of spirometry test carried out by pulmonologists, which has been demonstrated in previously published Italian and non-Italian studies as being one of the main critical issues concerning COPD to be addressed.

Not all patients with a COPD diagnosis could undergo spirometry as being bedridden, affected by dementia, or in general having difficulties in accessing pulmonary function labs, thus COPD diagnosis validation was not possible for all the COPD patients automatically identified in GPs’ archives. On the other hand whenever COPD validation was possible, our study demonstrated that misclassification of COPD in primary care databases is not trivial.

Ideally, two groups of comparable GPs practicing in the same catchment area should have been randomized to receive or less the educational intervention of COPD diagnosis and management. As this was an observational study carried out in naturalistic setting we used two different types of controls: (a) historical controls that is we compared the changes in COPD quality indicators of the same group of GPs participating to the study before and after receiving the intervention; (b) as results concerning historical controls may be biased by additional factors changing over time and having influence on COPD diagnosis and management, we considered, as additional comparator, a group of GPs unexposed to the educational intervention and who were part of the Health Search GP research networks. Both GPs participating to the Sicilian research network and those being part of Health Search belong to SIMG (Italian Scientific Society of General Practitioners) and, as such, receive in general comparable training in recording clinical information in GPs’ electronic archives. For this reason, we thought that this could be a suitable control to be effectively selected in naturalistic setting.

Another drawback of our study is the lack in lung function data that have been collected only for a sample of the studied subjects. These data could better describe the clinical condition of patients affected by COPD that are in charge of primary care. Therefore, actions should be planned to increase the awareness of GPs in systematically recording these information also in electronic health record systems.

Finally, although a cluster RCT represents the gold standard to demonstrate the effectiveness of an educational intervention, a pre-post design was performed due to pragmatic reasons. Indeed, in the real world practice, is impossible to guarantee that a specific group of GPs was not exposed to any other educational intervention and that there was none information sharing between exposed and unexposed GPs.

In conclusion, our study demonstrated that collaborative intervention programmes involving pulmonologists, clinical pharmacologist and GPs through periodic educational programs including remote continuous education and clinical audit may contribute to improve both diagnostic and therapeutic management of COPD in primary care settings, despite this effect may vary across GPs and indicators of COPD quality of care.

## Methods

### Study design and educational program

We carried out a 2 years prospective cohort study aimed to compare a set of COPD quality-of-care indicators before and after the educational program. In addition, frequency of COPD-related and all-cause hospitalizations over time of COPD patients, as outcome indicator, was measured during the study years (2013–2015) through linkage with the Sicilian Regional hospital discharge diagnosis database. The educational program consisted of periodic (every 6 months) and interactive face to face meetings (a full day for each meeting) in which two pulmonologists, clinical pharmacologists and other specialists provided all participating GPs with updated information on COPD diagnosis and pharmacological and non-pharmacological management and carried out training session on interpretation of spirometry findings. In addition, clinical audit through evaluation of several quality of care indicators over time and across GPs based on electronic data automatically extracted by GPs’ archives was performed.

Specifically, 15 quality-of-care indicators concerning COPD diagnosis, intervention on lifestyle, vaccinations and pharmacological therapies (Table 3) were re-evaluated against well-defined standards set on the principles of evidence-based medicine, to identify the required changes for COPD management improvement care. These indicators were developed and agreed initially by participating clinical pharmacologists, pulmonologists and GPs, on the basis of previously published reports of COPD patients^5,25^ as well as on specific loco-regional critical issues on COPD management. Results of each indicator were presented anonymously and discussed first during the meeting, while thereafter, each participating GPs received individual report with benchmarking of his/her performance for each quality of care indicators as compared to all participating GPs.

**Table 3:** List of quality of care indicators with relevant expected change after intervention

Indicator	Definition	Expected change	Possible reasons of expected change
Prevalence indicator			
1	COPD prevalence N. COPD patients (identified with a registration of ICD-CM-9: 496* or 491.2*) [numerator]/ total inhabitants of GPs participating to the study [denominator]	None	No change is expected in prevalence of COPD. The increase in newly occurring cases plus identification of false negative cases due to training on COPD diagnosis should be balanced by the death of the most severe cases and removal of false positive cases from the archives after the re-evaluation of previous diagnosis from GPs’.
Diagnostic process indicators			
2	% of COPD patients with at least one spirometry test registered lifetime N. COPD patients with at least one spirometry test registered lifetime [numerator]/ total COPD patients [denominator]	Increase	In agreement with GOLD guidelines spirometry test is required in the COPD diagnosis, Therefore, an increase in this data registration, especially in smokers, is expected.
3	% COPD patients, smokers or ex smokers, with at least one spirometry test registered lifetime N. COPD patients, smokers or ex smokers, with at least one spirometry test registered lifetime [numerator]/ total COPD patients, smokers or ex smokers [denominator]	Increase	
4	% COPD patients with at least one spirometry test registered in the last year N. COPD patients with at least one spirometry test registered in the last 365 days [numerator]/ total COPD patients [denominator]	Increase	
5	Mean number of spirometry registrations by COPD patients in the last two years N. spirometry registrations in the last two years [numerator]/ total COPD patients with at least one spirometry registration in the last two years [denominator]	Increase	
Preventive measures indicators			
6	% COPD patients with BMI registration lifetime N. COPD patients with BMI registration [numerator]/total COPD patients [denominator]	Increase	Overweight and obesity may modify the clinical overview of COPD, as well as comorbidities. Therefore, a careful registration and an increase of BMI data are expected.
7	% COPD patients with smoking registration lifetime N. COPD patients with smoking registration [numerator]/total COPD patients [denominator]	Increase	Tobacco smoke is the main risk factor for COPD. Therefore, a careful registration and an increase of smoking data are expected.
8	% COPD patients with influenza vaccination registration in the last year N. COPD patients with influenza vaccination registration in the last year [numerator]/total COPD patients [denominator]	Increase	In COPD patients, influenza vaccination can reduce serious illness and it should be offered in line with local guidelines. Therefore, a careful registration and an increase of influenza vaccination data are expected.
9	% COPD patients with pneumococcal vaccination registration in the last 4 years N. COPD patients with pneumococcal vaccination registration in the last 4 years [numerator]/total COPD patients [denominator]	Increase	GOLD guidelines recommend pneumococcal vaccination for COPD patients older than 64 years and those younger than 65 with and with predicted FEV_1_ < 40%. Therefore, a careful registration and an increase of pneumococcal vaccination data are expected.
Therapeutic process indicators			
10	% COPD patients with prescriptions for drugs targeting obstructive airway diseases in the last year N. COPD patients with ≥1 prescription for drugs targeting obstructive airway diseases in the last year/ total COPD patients [denominator]	Increase	When COPD diagnosis is confirmed, all patients should be treated chronically or as needed on the basis on GOLD staging. Therefore, an increase of prescriptions of drugs targeting obstructive airway is expected.
11	% COPD patients with at least one alone ICS prescription (without any ICS + LABA and/ or LAMA prescription) in the last year N. COPD patients with ≥1 ICS prescription as monotherapy (i.e., no LABA and/or LAMA prescription) in the last year [numerator] /total COPD patients [denominator]	Decrease	In COPD treatment, use of ICS is recommended only in combination with bronchodilators. Therefore, a decrease of ICS prescription as monotherapy is expected.
12	Occasional use (only 1 prescription) of long-acting bronchodilators in the last year N. COPD patients with only 1 prescription of long-acting bronchodilators plus ICS or less (LABA and/or LAMA and/or LABA/LAMA + ICS) in the last year [numerator]/ total COPD patients [denominator]	Decrease	Long-acting bronchodilators have to be used chronically. Therefore, a decrease of occasional use of long acting bronchodilators is expected.
13	% COPD patients in treatment with leukotriene receptor antagonists in the last year N. COPD patients with ≥1 prescription of leukotriene receptor antagonists in the last year [numerator]/ total COPD patients [denominator]	Decrease	Leukotriene receptor antagonists are not approved for COPD treatment A decrease of prescriptions of leukotriene receptor antagonists is expected.
14	% COPD patients with low adherence to LABA and/or LAMA therapy (±ICS) N. COPD patients with <8 packages of LABA and/or LAMA (±ICS) in the last year [numerator]/ total COPD patients in treatment with LABA and/or LAMA (±ICS) [denominator]	Decrease	Long acting bronchodilators are indicated for COPD patients as chronic treatment. Therefore, a decrease of patients with low adherence to LABA and/or LAMA (±ICS) therapy is expected.
15	% COPD patients with prolonged term therapy with ICS in monotherapy N. COPD patients with >5 packages of ICS (alone, without any ICS + LABA and/ or LAMA prescription) prescribed in the last year [numerator]/ total COPD patients in treatment with ICS (alone, without any ICS + LABA and/ or LAMA prescription)[denominator]	Decrease	Long term therapy with inhaled corticosteroid is associated with an increased risk of adverse effects (i.e. pneumonia, fractures). Therefore, a decrease of patients with prolonged term therapy with ICS is expected.

*BMI* body mass index, *ICS* inhaled corticosteroids, *LABA* long-acting beta agonist, *LAMA* long-acting muscarinic antagonist, *ICD9-CM* International Classification of Diseases, Ninth Revision, Clinical Modification

Furthermore, the program was supported by a continuous update and education on correct COPD diagnosis (e.g., careful interpretation of spirometry test by two pulmonologists) and therapy (based on GOLD recommendations^7^) which was further continued remotely via email. To improve communication among the coordinating center at the Academic Hospital ‘G. Martino’ of Messina and GPs, also a specific application for mobile (COPD APP) was developed and shared with GPs. This application allows GPs to view in real time their own values of each COPD quality of care indicators, to stage more easily COPD and to receive timely updates on COPD management by the coordinating center.

### Setting

Initially, 40 GPs members of the ‘Italian Society of General Practitioners’ (SIMG) practicing throughout Sicily agreed to participate to an educational intervention concerning COPD diagnosis and treatment, even if 7 were later on excluded from the analysis due to low quality of data extraction. Finally, 33 GPs caring a total population of 46,326 Sicilians participated to the study. Each GP enrolled in the study all COPD patients who were registered in their archives after patients had signed informed consent. Demographic and clinical data of these patients were extracted through software-specific dedicated and validated queries, fully anonymised and shared centrally with the coordinating centre, where data were stored, underwent quality check and were further analysed. The Ethical Committee of Academic Hospital “G. Martino” of Messina approved the study (Protocol N. 39–12, date 14 September 2012).

### Data collection

Data were collected in three different time-points: baseline (pre-intervention) vs. follow-up at 12 months and 24 months (post-intervention).

GPs performed extractions from their databases of all relevant information on patients with COPD diagnosis who were identified by searching in their electronic archives the specific International Classification of Diseases (ICD-9-CM (http://icd9.chrisendres.com/)) codes: 496*-‘chronic airway obstruction, not elsewhere classified’ and 491.2* - ‘obstructive chronic bronchitis’.

Data on demographic (age and gender), lifestyle, clinical and therapeutic characteristics were extracted for each patient.

Among lifestyle characteristics, cigarette smoking habits and body mass index (BMI) were collected.

The presence of the following comorbidities was evaluated by searching ICD-9-CM specific codes: diabetes mellitus, osteoporosis, anxiety and depression, heart failure, myocardial infarction, hypertension, dementia and pneumonia.

COPD pharmacological treatments were assessed on the basis of prescriptions of any drug for targeting obstructive airway diseases which were identified searching Anatomical Therapeutic Chemical (ATC) classification system code “R03*”.^26^ Specifically, respiratory drugs were categorized as follows: (a) inhaled corticosteroids (ICS) (ATC: R03BA*); (b) long-acting beta_2_ agonists LABA (ATC: R03AC*); (c) long-acting antimuscarinic antagonists (LAMA) (ATC: R03BB*); (d) LABA + ICS (ATC: R03AK*); (e) LABA/LAMA + ICS; (f) LABA + LAMA (ATC: R03AL*); (g) leukotriene receptor antagonists (ATC: R03DC*); (h) xanthines (ATC: R03DA*, R03DB*). As concomitant medications, the following drug classes were explored: antibiotics (ATC: J01A*, J01C*, J01DB*, J01DC*, J01F*, J01MA*), anti-thrombotic drugs (ATC: B01AA03, B01AB*), antihypertensive drugs (ATC: C02*, C03*, C07*, C08*, C09A*, C09B*), systemic corticosteroids (ATC: H02*) and immunosuppressive drugs (ATC: L04A*). Information on influenza vaccination and anti-*Streptococcus pneumoniae* vaccination was also derived from electronic medical records.

### Assessment of educational intervention effectiveness

The effectiveness of educational intervention was evaluated by comparing the values of the 15 quality-of-care indicators at baseline (before intervention) and in two different follow-up time points (at 12 and 24 months after the intervention). These indicators were grouped into several types: COPD diagnostic process, preventive measures and therapy. For each indicator, the effect of the intervention was evaluated by comparing the values of the participating GPs before and after the intervention (Table 4).

**Table 4:** Comparison of mean values of GP’s individual quality of care indicators before and after the educational intervention and vs. Italian general population (Health Search—IMS Health Longitudinal Patient Database: HSD)

Indicator	Pre-intervention	Post-intervention		Health Search	*P* value^a^		Achievement of intervention goal	ICC^b^
	Baseline	FUP at 12mo.	FUP at 24mo		FUP at 12 mo vs Baseline	FUP at 24 mo vs. Baseline		
	% [num./denom.]	% [num./denom.]	% [num./denom.]	% [num./denom.]				
Prevalence indicator								
1. COPD prevalence	3.2 [1465/46,326]	3.0 [1395/46,326]	3.0 [1388/46,620]	3.0 [31,691/1,054,376]	—	—	—	—
Diagnostic process indicators								
2. Spirometry lifetime	59.7 [875/1465]	70.5 [984/1395]	73.0 [1013/1388]	64.8 [20,524/31,691]	<0.001	<0.001		0.243
3. Spirometry lifetime among smokers	70.5 [548/777]	77.7 [617/794]	81.0 [657/811]	65.3 [11,061/16,931]	<0.001	<0.001		0.185
4. Spirometry in the last year	25.3 [371/1465]	36.1 [504/1395]	32.1 [445/1388]	13.6 [4070/29,881]	0.002	0.083		0.134
5. Mean n. spirometry in the last two years	1.9 [1039/537]	2.5 [1664/656]	3.2 [2162/681]	1.7 [10,771/6431]	<0.001	<0.001		0.064
Preventive measures indicators								
6. BMI registration lifetime	75.8 [1110/1465]	81.1 [1131/1395]	81.7 [1134/1388]	76.4 [24,228/31,691]	<0.001	<0.001		0.312
7. Smoking registration lifetime	76.5 [1120/1465]	80.2 [1119/1395]	81.0 [1124/1388]	80.2 [25,415/31,691]	0.002	<0.001		0.583
8. Influenza vaccination in the last year	57.2 [838/1465]	55.1 [769/1395]	55.7 [773/1388]	31.0 [9262/29,881]	0.540	0.989		0.367
9. Pneumococcal vaccination in the last 4 years	32.6 [477/1465]	34.8 [485/1395]	35.1 [487/1388]	15.0 [3594/23,920]	0.090	0.062		0.334
Therapeutic process indicators								
10. Drugs targeting obstructive airway diseases in the last year	61.0 [893/1465]	64.7 [903/1395]	63.3 [879/1388]	53.2 [15,903/29,881]	0.028	0.795		0.042
11. ICS in monotherapy in the last year	9.6 [138/1465]	10.3 [144/1395]	9.9 [137/1388]	7.7 [2306/29,881]	0.923	0.478		0.085
12. Occasional use of LABA and/or LAMA (±ICS) in the last year	8.5 [125/1465]	8.1 [113/1395]	6.3 [87/1388]	4.5 [1347/29,881]	0.894	0.047		0.061
13. Leukotriene receptor antagonists use in the last year	2.3 [34/1465]	1.7 [24/1395]	1.9 [27/1388]	1.8 [539/29,881]	0.016	0.049		0.173
14. Low adherence to LABA and/or LAMA (±ICS)	61.6 [424/688]	56.9 [400/703]	54.7 [378/690]	55.0 [6718/12,213]	0.105	0.060		0.136
15. Prolonged use of ICS	5.8 [8/138]	7.6 [11/144]	5.1 [7/137]	9.3 [216/2306]	0.340	0.218		0.222

*BMI* body mass index, *ICS* inhaled corticosteroids, *LABA* long acting beta agonist, *LAMA* long acting muscarinic antagonist, *ICD-9-CM* International Classification of Diseases, Ninth Revision, Clinical Modification

^a^*p*-values from hierarchical generalized linear models, accounting for clustering due to GPs

^b^*ICC* intra-cluster correlation at baseline

The educational program addressed substantially the criteria to be considered for COPD diagnosis, as indicated in the international guidelines, also through training session on spirometry test interpretation. As a consequence of the educational program, GPs revaluated the diagnosis of patients with a registered diagnosis of COPD if a spirometry test was not available.

As diagnostic process indicators, the rate of subjects with at least a spirometry test (anytime and in the last year) was considered. Spirometry test was considered as the reference indicator for which computing a power calculation. A total sample size of 1465 patients with COPD diagnosis, belonged into the intervention group from the Sicilian GP research network, achieves 97.8% of statistical power and a significance level of 5% (type I error) to detect a minimum difference in the proportions of patients with a registration of a spirometry test lifetime between two paired groups (evaluated at baseline and after 24 months from baseline) of 15%, using McNemar’s two-sided test. Power calculation also accounted for the ‘design effect’ due to the clustered nature of the data (i.e., patients within GPs) having assumed: an intra-cluster correlation (ICC) of 0.24 for spirometry lifetime at baseline, an average cluster size of 44 patients enrolled per GP and a proportion of patients having discordant registration status in spirometry lifetime between baseline and 24 months of 20% (i.e., patients who did not have a spirometry lifetime registration at baseline but get this one after 24 months).

The rates of influenza and *Streptococcus pneumoniae* vaccinations as well as and the rates of smoking and BMI registration for COPD patients in GPs’ archives were evaluated as preventive measures indicators.

To measure the appropriateness of pharmacological therapy, in line with GOLD recommendations, first, the rate of patients under treatment (i.e., percentage of COPD patients with at least a prescription of any drug targeting obstructive airway diseases in the observation year) was measured. Moreover, we considered as inappropriate COPD treatment: (a) use of ICS as monotherapy; (b) prolonged ICS therapy (i.e., >5 consecutive ICS packages within one year); (c) occasional use (i.e., a single prescription within 1 year) of LABA/LAMA; and (d) low adherence to LABA and/or LAMA (+ICS or less) (i.e., <8 bronchodilators packages within 1 year); and (e) use of leukotriene receptor antagonists (i.e., ≥1 prescription within 1 year).

The values of these indicators recorded among Sicilian GPs were compared also with a cohort of GPs distributed all over Italy, by using the Health Search—IMS Health Longitudinal Patient Database (HSD).^24^ This database contains clinical records of more than 1.1 million Italian persons in charge of 800 GPs. This data source is widely used to conduct observational research in the primary care setting, as previously demonstrated.^27^

Data derived from hospitalizations are showed in Fig. 2. Moreover, for each indicator we performed benchmarking of average values across different GPs (Supplementary Material).

### Statistical analysis

Patient characteristics were reported as median along with interquartile range (i.e., first-third quartiles) or absolute frequency and percentages for continuous and categorical variables, respectively. The only continuous variable was represented by patient’s age, which distribution significantly deviated from Normal (evaluated by means of Shapiro–Wilk’s test, Kolmogorov–Smirnov’s test and Q-Q plot).

COPD prevalence was calculated per 100 inhabitants by dividing the number of patients with a COPD diagnosis with the number of subjects alive and registered in the GPs’ lists.

Comparisons between paired patients’ groups (baseline vs after 24 months) with COPD diagnosis were performed using Mc Nemar’s test for all categorical variables. This test was not performed for the evaluation of patient’s sex and age changes over time because it is tautological that patient’s sex cannot change from baseline, whilst patient’s age obviously increase of 2 years exactly from baseline. Comparisons between HSD and patients’ group at 24 months were performed using Chi-square test, while Student’s test was used for median values.

For each quality-of-care indicator, the ‘crude’ prevalence of COPD patients with the registered indicator was calculated at baseline and after 12 and 24 months after the intervention as stated above. Moreover, the ‘adjusted for GPs clustering’ prevalence of COPD patients with the registered indicator was estimated at baseline and after 12 and 24 months after the intervention from hierarchical generalized linear models (HGLM) for longitudinal data and pairwise comparisons between paired adjusted prevalence at 12 and 24 months were performed with respect to the baseline. Within this framework, the logistic distribution was assumed to model the distribution of such proportions and the follow-up time was included as categorical covariate (with random intercept and slopes).

The first-order autoregressive covariance structure was used to account for the correlation between repeated measurements over time. Estimated proportions were reported along with their 95% confidence interval (95% CI). Moreover, the ICC coefficient at baseline was estimated for each quality-of-care indicator, separately. Longitudinal plots of the estimated mean proportions over time (i.e., which accounted for GPs clustering effect) were further reported along with error bars which represented 95% CI. Within each longitudinal plot, profiles estimated by all GPs (i.e., trajectories) were further represented. As the mean proportions changed at each time point at issue (i.e., baseline, 12 and 24 months), each GP was classified (for each quality-of-care indicator and for each time point, separately) to belong into the group of those who were: (1) ‘below the mean’ if his/her estimated proportion fall below the lower bound of the estimated 95% CI around the mean; (2) ‘within the mean’ if his/her estimated proportion was included into estimated 95% CI around the mean; (3) ‘above the mean’ if his/her estimated proportion fall above the upper bound of the estimated 95% CI around the mean (Table 2). According to such classification: a ‘negative’, ‘neutral’ or ‘positive’ attitude and behaviour of each GP for each specific indicator can be deduced with respect to the type of indicator (i.e., a negative GPs’ attitude does not necessarily mean that the GP was classified ‘below the mean’ for the specific indicator). Therefore, the following two frequency distributions were derived: (1) the distribution of GPs (%) with ‘negative’, ‘neutral’ and ‘positive’ behaviour for each specific indicator at the three time points; (2) the distribution of indicators (%) at which GPs achieved ‘negative’, ‘neutral’ and ‘positive’ behaviours at the three time points, separately. Histograms of such distributions were further reported in Supplementary Material.

Two-sided *p*-values < 0.05 were considered for statistical significance. All analyses were performed using SAS Software, Release 9.4 (SAS Institute, Cary, NC, USA) and ggplot2 package of R version 3.3.2 (http://www.r-project.org).

Methods were performed in accordance with relevant regulations and guidelines.

### Data availability

Only aggregated data are available based on agreement with SIMG. It is not possible to share any independent patient level data.

## Consortia: GPs participating to the Sicilian COPD research team, and belonging to the Sicilian section of Italian college of general practitioners:

Amato Salvatore, Aulicino Caterina, Beneventano Guglielmo, Caccamo Orazio Antonio, Campo Francesco, Campo Salvatore, Caroppo Michela, Claudio Sergio, Consiglio Girolamo, Crescenti Angelo, Crescenti Francesco, D’Amico Filippo, Di Carlo Vittorio, Di Geronimo Luciana, Di Giacomo Giovanni, Di Gregorio Carmelo, Di Guardo Antonino, Di Maggio Edoardo, Di Silvestre Baldassare, Fasulo Serena, Gambino Rosa, Giardina Giovanni, Gurgone Francesco, Imburgia Giovanni, Inferrera Santi, Iraci Tindaro, La Mattina Salvatore Carmelo, La Verde Francesco, Lipari Luigi, Lombardo Francesco Paolo, Mangione Marcello, Marino Sebastiano, Mastrosimone Giuseppe, Maurici Vincenzo, Merlino Giovanni, Milazzo Vito, Oddo Salvatore, Paradisi Vincenza, Pasqualetto Salvatore, Pozzecco Umberto, Quartetti Giovanni, Saccà Felice, Salamone Francesco, Salvo Anna, Santoro Giuseppe, Schifano Giuseppe, Simonetti Maria Teresa, Troisi Rosina, Zafonte Rita

## Electronic supplementary material

Supplemental Material(DOCX 2238 kb)
