# Supplementary material for: Improvement in the management of chronic obstructive pulmonary disease following a clinical educational program: results from a prospective cohort study in the Sicilian general practice setting
Source: NPJ Prim Care Respir Med. 2018 Mar 23;28:10. doi: 10.1038/s41533-018-0077-7 (PMC5865126; doi:10.1038/s41533-018-0077-7)

**Supplementary material**

**Figure 1** GP- and time period-specific prevalence and diagnostic process indicators


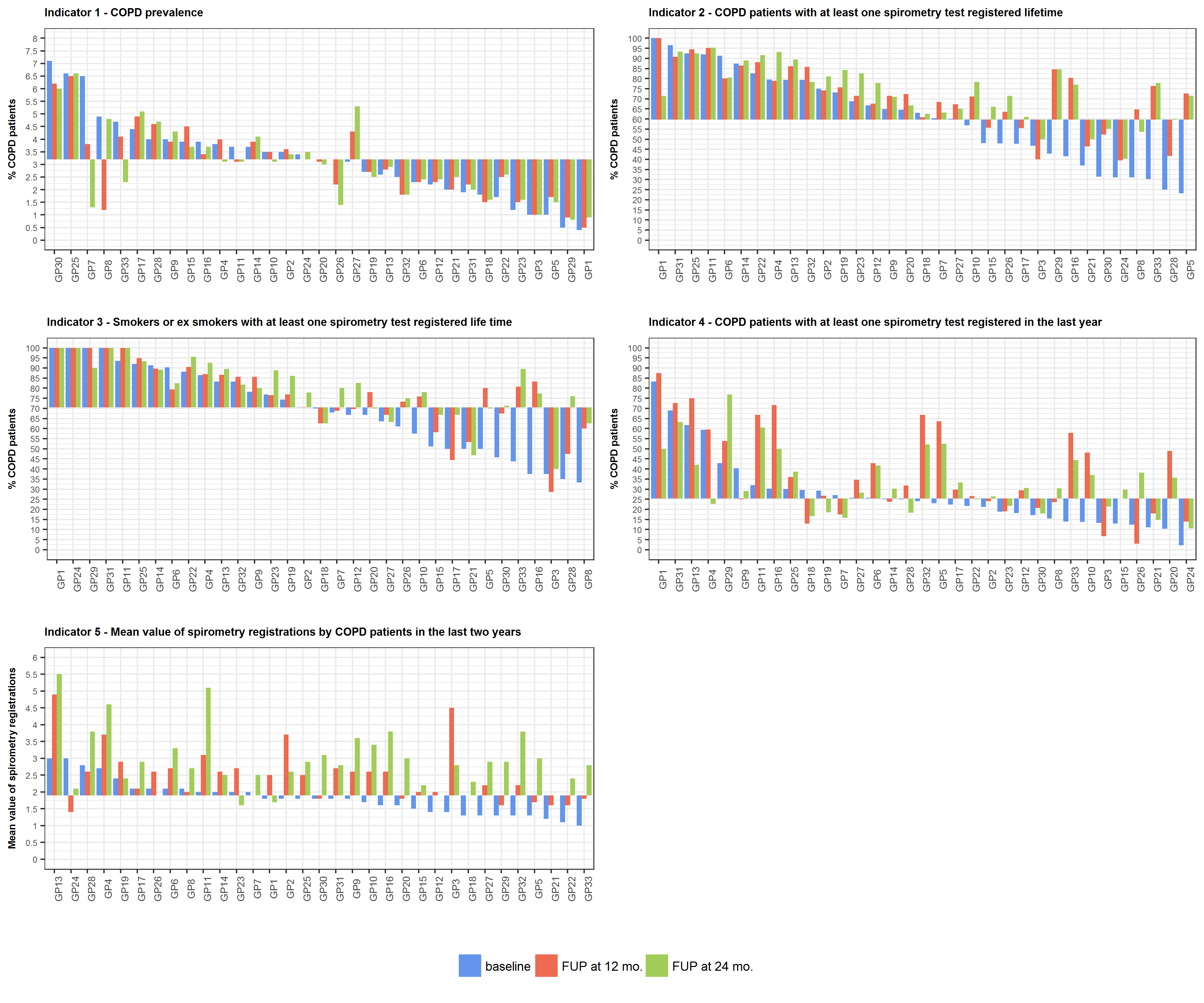


**Figure 2.** GP- and time period-specific preventive measures indicators


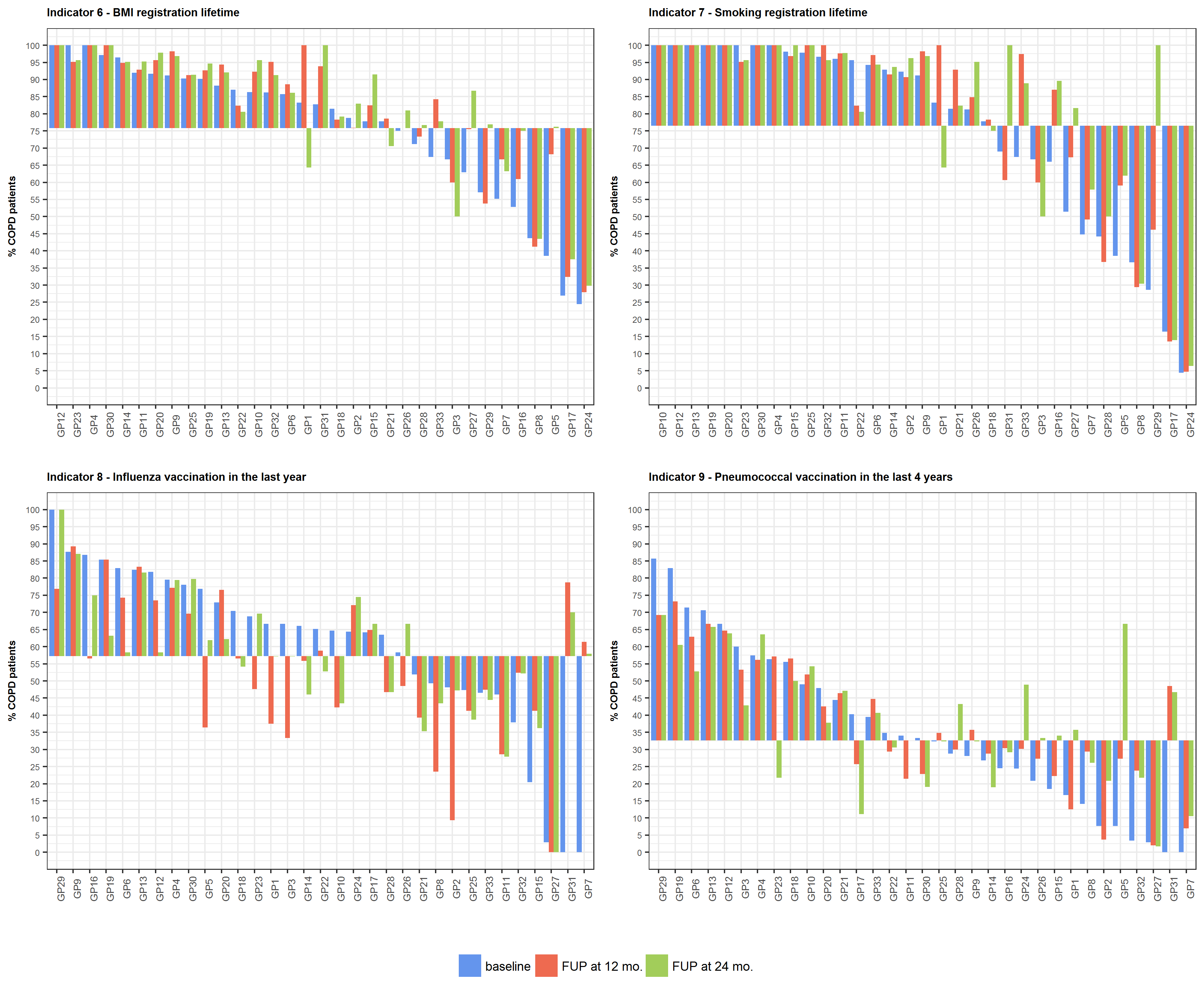


**Figure 3.** GP- and time period-specific therapeutic process indicators


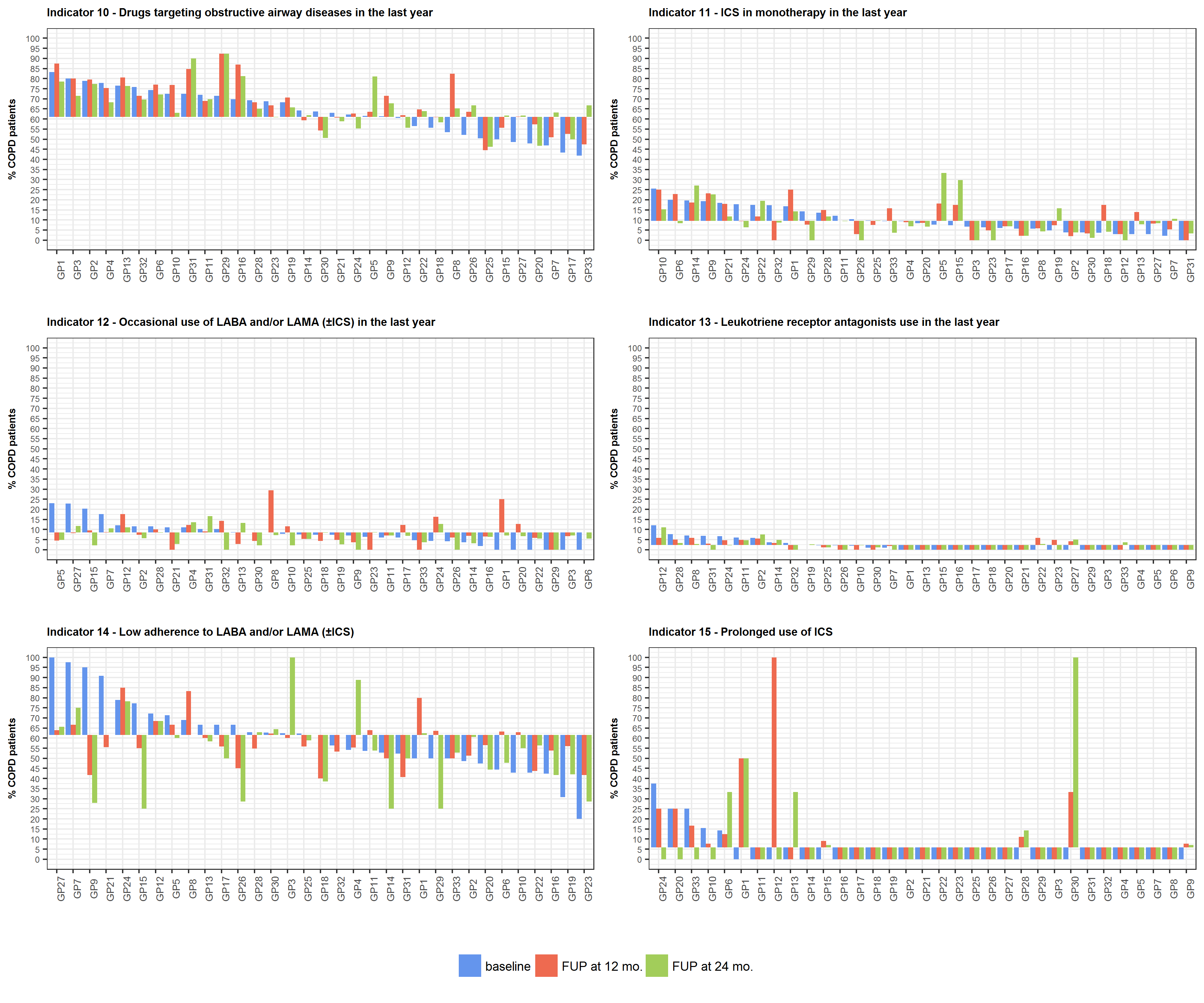


**Figure 4.** Distribution of indicators with negative, neutral and positive behavior (%) for each GP at the three time points
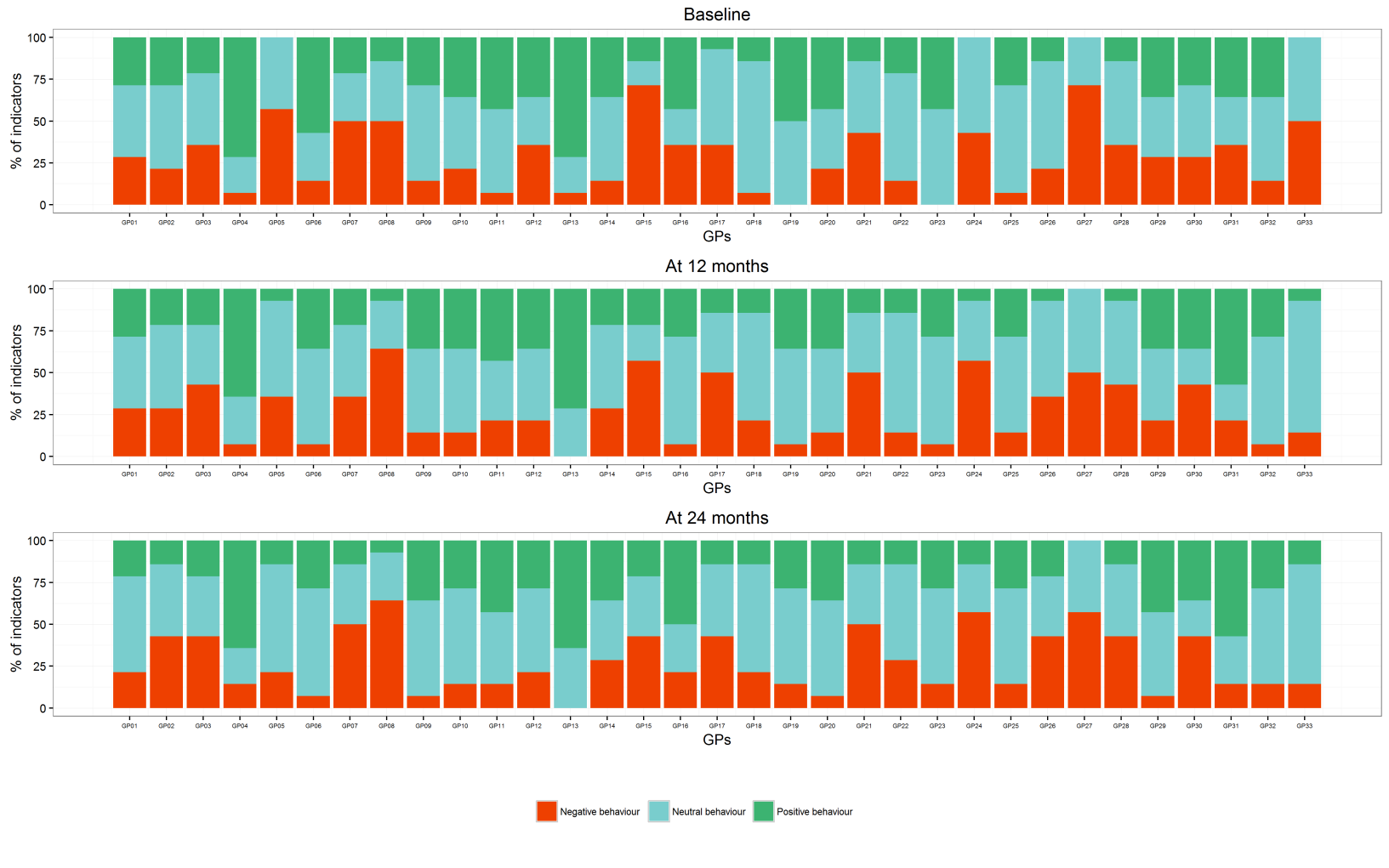

Supplement: Supplementary file 1 — Supplemental Material(DOCX 2238 kb) [file 41533_2018_77_MOESM1_ESM.docx]
